# Limbic control over the homeostatic need for sodium

**DOI:** 10.1038/s41598-018-37405-w

**Published:** 2019-01-31

**Authors:** Jeroen P. H. Verharen, Theresia J. M. Roelofs, Shanice Menting-Henry, Mieneke C. M. Luijendijk, Louk J. M. J. Vanderschuren, Roger A. H. Adan

**Affiliations:** 1 Brain Center Rudolf Magnus, Department of Translational Neuroscience, University Medical Center Utrecht, Utrecht, The Netherlands; 2 Department of Animals in Science and Society, Division of Behavioural Neuroscience, Faculty of Veterinary Medicine, Utrecht University, Utrecht, The Netherlands; 3 Institute of Physiology and Neuroscience, Sahlgrenska Academy at the University of Gothenburg, Gothenburg, Sweden

## Abstract

The homeostatic need for sodium is one of the strongest motivational drives known in animals. Although the brain regions involved in the sensory detection of sodium levels have been mapped relatively well, data about the neural basis of the motivational properties of salt appetite, including a role for midbrain dopamine cells, have been inconclusive. Here, we employed a combination of fiber photometry, behavioral pharmacology and c-Fos immunohistochemistry to study the involvement of the mesocorticolimbic dopamine system in salt appetite in rats. We observed that sodium deficiency affected the responses of dopaminergic midbrain neurons to salt tasting, suggesting that these neurons encode appetitive properties of sodium. We further observed a significant reduction in the consumption of salt after pharmacological inactivation of the nucleus accumbens (but not the medial prefrontal cortex), and microstructure analysis of licking behavior suggested that this was due to decreased motivation for, but not appreciation of salt. However, this was not dependent on dopaminergic neurotransmission in that area, as infusion of a dopamine receptor antagonist into the nucleus accumbens did not alter salt appetite. We conclude that the nucleus accumbens, but not medial prefrontal cortex, is important for the behavioral expression of salt appetite by mediating its motivational component, but that the switch in salt appreciation after sodium depletion, although detected by midbrain dopamine neurons, must arise from other areas.

## Introduction

In order to obtain all nutrients necessary for survival, organisms need to make adaptive food choices based on their homeostatic needs^1,2^. For example, when an organism’s body senses a shortage of a certain nutrient, it may, consciously or not, choose foods that will replenish this need^2,3^. Of all the nutrients, a deficiency in sodium is one of the strongest homeostatic drives known in animals, evoking intense cravings for salty foods after salt deprivation, which has been consistently reported in a wide range of species^4,5^. Although sodium is abundant in modern Western diets, it is relatively scarce in natural resources, which has likely contributed to the development of this homeostatic drive^6,7^.

A remarkable observation that illustrates the innate drive for sodium is that rats normally experience a hypertonic sodium solution as aversive, but that this solution is experienced as positive and consumed in high amounts when rats are low on sodium, a phenomenon known as salt appetite^4,5,8,9^. Such a switch in the experience of a flavor from aversive to appetitive, driven by a homeostatic need, is a prime example of how adaptive the interaction between sensory and reward systems can be in order to maintain homeostasis and ensure survival. Elucidating the mechanisms that underlie this switch may therefore provide interesting insights into the flexibility of brain circuits that mediate reward.

A variety of brain areas has been shown to be involved in salt appetite. Not surprisingly, this includes brain structures involved in the sensory processing of taste, such as the parabrachial nucleus^10^ and the nucleus of the solitary tract^11,12^. Other brain areas implicated in salt appetite are the lateral and paraventricular nucleus of the hypothalamus, the preoptic area, the subfornical organ, the central amygdala and the bed nucleus of the stria terminalis (for a review see ref.^13^). Given its role in processing rewarding and aversive stimuli^14,15^, a logical candidate for the mediation of salt appetite is the mesocorticolimbic dopamine (DA) system, consisting of DA neurons in the ventral tegmental area (VTA) projecting to the nucleus accumbens (NAc) and medial prefrontal cortex (mPFC). However, data about the involvement of this circuit in salt appetite has been inconclusive. On the one hand, a total ablation of the VTA^16^ or DA terminals in the entire brain^17^, as well as the infusion of DA receptor agonists or antagonists in the nucleus accumbens^18^ does not affect salt appetite, suggesting that motivation for salt bypasses the mesoaccumbens DA pathway. On the other hand, it has been observed that infusion of a delta-opioid receptor antagonist into the VTA decreases salt appetite^18^, and that a sodium-depleted state is associated with decreased DA transporter activity^19^ and altered spine morphology^20^ in the nucleus accumbens. A recent study demonstrated, using fast-scan cyclic voltammetry, that tasting a sodium solution evoked phasic dopamine release in the rat nucleus accumbens shell after sodium deprivation, but not under normal conditions^21^. Furthermore, this study showed that hindbrain neurons projecting to the VTA displayed increased c-Fos expression after salt deprivation. Another recent study showed that optogenetic or chemogenetic activation of VTA DA neurons in mice reduced intake of a high-concentration (but not low-concentration) salt jelly, while chemogenetic inhibition of these same neurons had no effect on salt intake^22^.

In this study, we attempted to further elucidate the role of the mesocorticolimbic DA system in salt appetite. Towards this aim, we combined fiber photometry, behavioral pharmacology and c-Fos immunohistochemistry to study *in vivo* VTA DA neuron dynamics during sodium deficiency, and the importance of the NAc and mPFC, the two major output regions of these neurons, for salt appetite. By employing a microstructural analysis of licking behavior, we tried to discern effects of manipulations of the mesocorticolimbic system on the motivation for versus the appreciation of salt. We hypothesized that VTA DA neurons may respond differently to salty solutions during a normal versus sodium-depleted state, and that these changes in DA cell responsiveness are important for the expression of behaviors associated with salt appetite.

## Results

### No changes in c-Fos expression in DA nuclei after sodium deprivation

**Figure 1:**
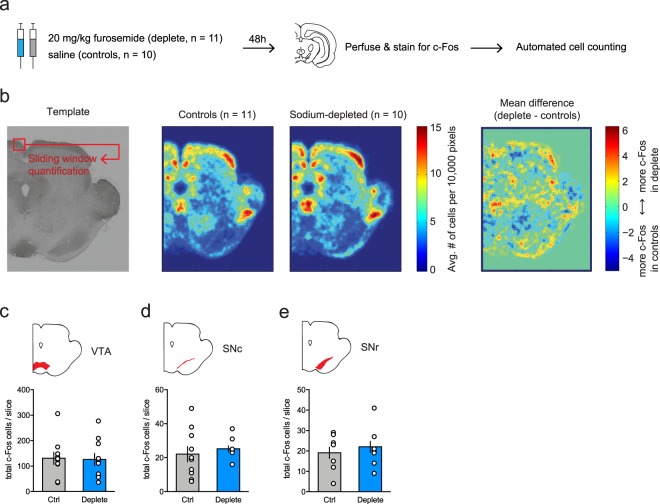
c-Fos analysis of midbrain slices after sodium deprivation. (**a**) Experimental design. (**b**) From left to right: a coronal slice of the midbrain that included the VTA and substantia nigra was used to create a template on which the other midbrain slices were overlayed in order to perform whole-slice automated cell counting – average c-Fos density in control animals (n = 11) – average c-Fos density in sodium-depleted animals (n = 10) – mean difference in c-Fos expression between controls and depleted animals indicating similar levels of c-Fos expression. (**c**–**e**) Region-of-interest analysis showed no significant differences in the number of c-Fos positive cells in the VTA (**c**; t_18_ = 0.15, p = 0.88), the substantia nigra pars compacta (SNc; **d**; t_18_ = 0.64, *P* = 0.53), or the substantia nigra pars reticulata (SNr; **e**; t_18_ = 0.75, *P* = 0.46).

In an attempt to substantiate the findings of ref.^22^, that showed that sodium deprivation did not change baseline activity of VTA DA neurons, we analyzed c-Fos immunoreactivity in a coronal slice of the midbrain that included the VTA and substantia nigra (Fig. 1a; *n* = 21). Based on a typical brain slice, we created a template on which we overlayed all the other slices in order to perform whole-slice automated cell counting. A visual sliding-window analysis revealed fairly similar levels of c-Fos expression between animals in a sodium-depleted state (induced by treatment with the diuretic furosemide; see Methods) versus a control state around these nuclei (Fig. 1b). Indeed, region-of-interest analysis showed no significant differences in the number of c-Fos positive cells in the VTA (Fig. 1c), the substantia nigra pars compacta (SNc; Fig. 1d), or the substantia nigra pars reticulata (SNr; Fig. 1e). Together, these data support the finding that baseline activity of neurons in midbrain DA nuclei was not altered by sodium deprivation.

### Dopamine neurons encode a switch in sodium appreciation

**Figure 2:**
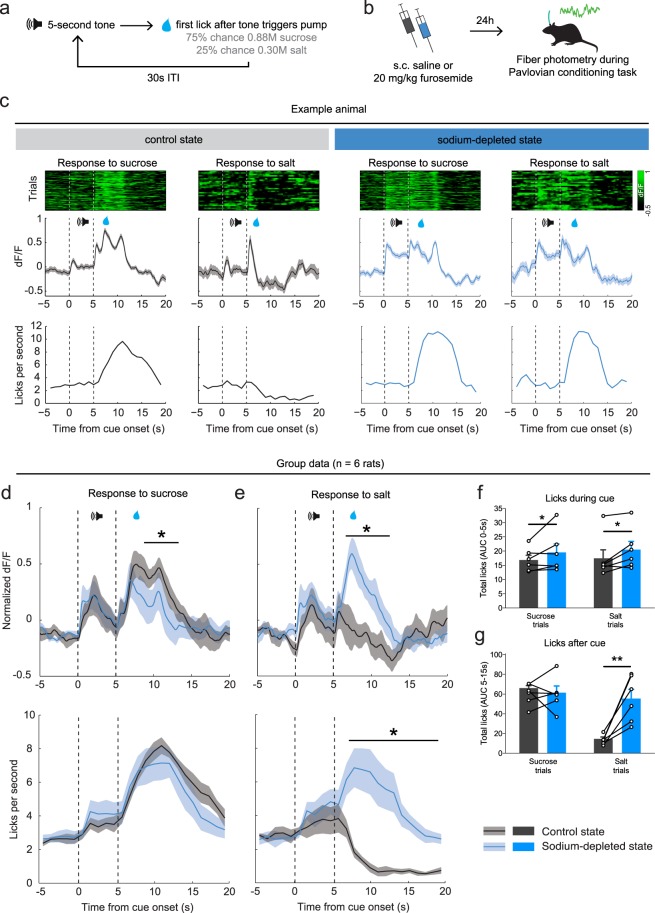
*In vivo* fiber photometry of VTA DA neurons during sodium depletion. (**a**) The Pavlovian conditioning task that was used for *in vivo* fiber photometry consisted of a five-second auditory tone followed by delivery of a nutritional solution, being either a sucrose solution (in 75% of trials) or a NaCl solution (in 25% of trials). A 30-second inter trial interval (ITI) separated the trial from the next auditory tone. (**b**) Animals were tested twice; once after a subcutaneous (s.c.) saline injection, i.e. a control state, and once after a furosemide injection, i.e. a sodium-depleted state. Rats were tested after being in a specific state for 24 hours. (**c**) Population responses of VTA DA neurons of an example animal (sampling rate 100 Hz). Shown are the control state (left) and sodium-depleted state (right). Reward was delivered for 5 s after the first lick after cue offset (5 s). (**d**) (top panel) Sodium depletion decreased VTA DA neuron responses to sucrose (2-way repeated measures ANOVA, main effect of treatment, F_1,5_ = 2.494, p = 0.1751; treatment × time interaction effect, F_2499,12495_ = 1.335, *P < 0.0001; post-hoc test significant between 7.9–13.1 s post-stimulus). (bottom panel) ANOVA revealed no differences in the number of licks for sucrose between the two treatments (main effect of treatment, F_1,5_ = 1.307, p = 0.3046; treatment × time interaction effect, F_23,115_ = 0.9798, *P* = 0.4963). (**e**) (top panel) Mean responses of all animals to salt indicated that sodium depletion increased VTA DA neuron response to salt (ANOVA, main effect of treatment, F_1,5_ = 9.463, *P* = 0.0276; treatment × time interaction effect, F_2499,12495_ = 2.188, *P < 0.0001; post-hoc test significant between 6.8–12.9 s post-stimulus). (bottom panel) Sodium depletion increased the number of licks for salt (ANOVA, main effect of treatment, F_1,5_ = 10.13, *P* < 0.0001; treatment × time interaction effect, F_23,115_ = 10.13, *P = 0.0016; post-hoc Sidak’s test, significant 7–18 s post-stimulus). (**f**) Salt depletion increased the number of licks during the 5-second cue across both trial types (ANOVA, main effect of treatment, F_1,5_ = 10.10, *P = 0.0246; but no treatment × tastant interaction effect, F_1,5_ = 0.03624, *P* = 0.8565; and no main effect of tastant, F_1,5_ = 0.6428, *P* = 0.4591). (**g**) Salt depletion increased the number of licks for salt, but not for sucrose (ANOVA, treatment × tastant interaction effect, F_1,5_ = 19.93, *P* = 0.0066; post-hoc Sidak’s test, control vs depleted state: sucrose t_5_ = 0.017, *P* = 0.9998; salt t_5_ = 6.297, **P = 0.0030).

To study how VTA DA neurons respond to the taste of salt during normal and low levels of sodium in the body, we injected a viral vector carrying Cre-dependent GCaMP6s into the VTA of TH::Cre rats (Supplementary Fig. 1) and measured VTA DA neuron dynamics using fiber photometry during a Pavlovian conditioning task. In this task (Fig. 2a), rats (*n* = 6) learned that a 5-second auditory tone preceded the delivery of a nutritional solution, which was usually a tasty sucrose solution (3 out of 4 trials), but sometimes a NaCl solution (1 out of 4 trials). We tested the responses of the animals to these solutions on two occasions: once in a sodium-deficient state, 24 h after injection with furosemide, and once under baseline conditions (Fig. 2b).

In the control state, animals vigorously licked for sucrose, but refrained from licking when a sodium solution was delivered, in line with our expectations that high-sodium concentrations are aversive to rats. Accordingly, VTA DA neuron population activity increased during the consumption of sucrose, while the delivery of salt resulted in sub-baseline levels of DA neuron activity. This is illustrated in both an example animal (Fig. 2c, left panel) as well as on a group level (Fig. 2d,e).

In the sodium-depleted state, animals showed increased licking during the reward-preceding cue, although this effect was numerically modest (Fig. 2f). In contrast, sodium depletion strongly increased the number of licks during the delivery of the salty solution, while the number of licks for sucrose was not dependent on the sodium state of the animals (Fig. 2g). The difference in licking for salt after sodium deprivation extends beyond the peaks in DA neuron activity, and this is especially driven by a long attenuation of licking behavior after salt exposure in the control state (Fig. 2e, bottom panels). In line with this appreciation of salt after sodium deprivation, we observed increased levels of VTA DA neuron activity in response to salt delivery (Fig. 2e), with responses that were even higher than those after sucrose delivery (compare Fig. 2d,e, blue curves). Although numerically more modest than the changed DA neuron responsiveness to salt, we observed a lower DA neuron activation to sucrose during a salt-depleted state compared to the control state (Fig. 2d). Importantly, we observed no changes in fluorescent activity in animals that were injected with an activity-independent control fluorophore (n = 4; Supplementary Fig. 2), indicating that the observed fluorescent signals were driven by neuronal activity.

In sum, we show that a salty solution is considered aversive by rats under normal conditions, as shown by the termination of licking behavior and sub-baseline levels of VTA DA neuron activity, but that this same solution is considered appetitive in a sodium-depleted state, accompanied by vigorous licking for salt and large peaks in DA neuron activity.

### Inactivation of NAc, but not mPFC, diminished drinking behavior without affecting salt appetite

**Figure 3:**
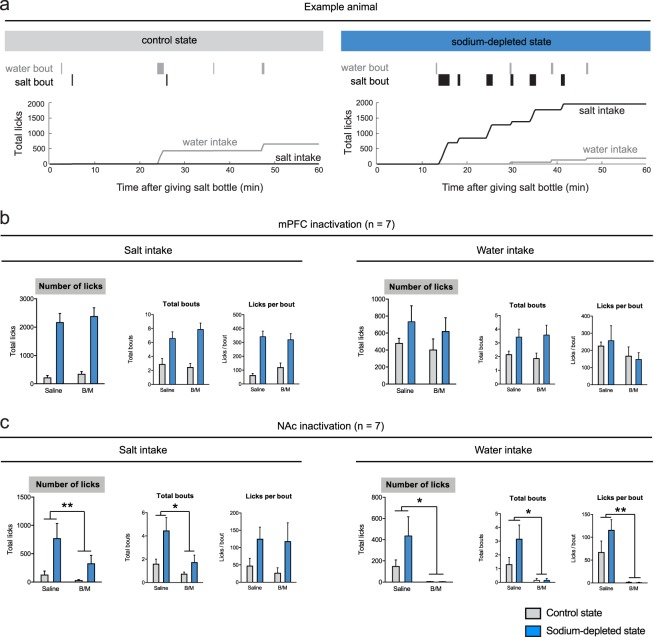
Effects of pharmacological inactivation of VTA target regions on salt appetite. (**a**) Microstructure analysis of licking behavior in an example animal once in a control state (left) and once in a sodium-depleted state (right). At time = 0 min the salt bottle was given back to the animal and its drinking behavior was analyzed as number of licks (grey line for water intake, black line for salt intake). On the upper part of the graph, bout analyses for salt and water intake shows frequency and length of the bouts. (**b**) Effect of mPFC inactivation on salt intake (left) and water intake (right). No main effect of mPFC inactivation by baclofen and muscimol (B/M) or interaction effect was detected. (**c**) Effect of NAc inactivation on salt intake (left) and water intake (right). Inactivation of the NAc decreased sodium intake, which was driven by a decrease in the number of licking bouts. A significant main effect of state was detected for the number of sodium licking bouts, and a trend towards an effect of sodium depletion on the number of licks and bout size. No B/M × state interaction effects were observed. Inactivation of the NAc also abolished water consumption, as a main effect of B/M was found on the number of water licks, driven by effects on the number of bouts and licks per bout. A single asterisk annotation per graph indicates a main effect of B/M; see also the Supplementary statistics table. **P < 0.01, *P < 0.05

To investigate the behavioral structure of salt appetite, we assessed free intake of a 0.45 M NaCl solution, as well as intake of demineralized water, by using mechanical lickometers present in the animals’ home cages, which measured the numbers of licks per 12 s bins (in a within-subjects, counterbalanced design; each animal was tested four times). To gain insight into the appetitive components of sodium appetite, we performed a microstructure analysis of licking behavior, calculating the number of licking bouts that animals made, as well as the size of each of these bouts (Fig. 3a). As expected, animals that were brought into a sodium-depleted state consistently consumed more of the sodium solution, which was driven by an increase in the frequency as well as the size of licking bouts (see Figs 3 and 5). Note that these animals had *ad libitum* access to demineralized water, but had no access to salt in the 24 h prior to the measurements.

To study the role of the two main VTA DA neuron output regions, the mPFC and NAc, in the regulation of salt appetite, we pharmacologically inactivated the mPFC and the NAc using micro-infusions of a mixture of the GABA receptor agonists baclofen and muscimol (B/M). Rats were brought in a sodium-depleted or a control state for 24 h, after which they received infusions with either B/M or saline. Subsequently, animals received a 0.45 M NaCl solution, in addition to a bottle of demineralized water that was already present in the cage.

We first assessed salt appetite upon mPFC inactivation (*n* = 7; Supplementary Fig. 3a). A two-way repeated measures ANOVA revealed increased consumption of the sodium solution in sodium-depleted animals (main effect of state), which was driven by an increase in the frequency of licking bouts as well as by the size of these bouts (Fig. 3b, left panels). Inactivation of the mPFC, however, did not impact consumption of the sodium solution (Fig. 3b, left panels), nor of demineralized water (Fig. 3b, right panels), as the ANOVA revealed no main effect of B/M or B/M × state interaction effect.

Inactivation of the NAc (*n* = 7; Supplementary Fig. 3b,c) significantly decreased sodium intake, as the two-way repeated measures ANOVA revealed a main effect of B/M on the licks for salt, which was driven by a decrease in the number of licking bouts but not by the size of these bouts (Fig. 3c, left panels). However, in a sodium-depleted state, animals still drank a substantial amount of salt, even after B/M infusion (on average 323 ± 148 s.e.m. licks in the 1 h recording session). Indeed, there was a significant main effect of sodium depletion (state) on the number of sodium licking bouts, and a trend towards an effect of sodium depletion on the number of licks and bout size. Importantly, no B/M × state interaction effects were observed, indicating that the effects of sodium deprivation on salt intake were still present after NAc inactivation, although numerically more modest.

Licking for water in sodium-deprived and control rats also decreased upon infusion of B/M into the NAc, as a significant main effect of B/M was observed (Fig. 3c, right panels). In contrast to licking for salt, water consumption was almost fully abolished in both groups of rats (on average 4 ± 1–2 s.e.m. licks in the 1 h recording session), without a main effect of state or B/M × state interaction effect. Collectively, these data show that inactivation of the NAc decreases intake of salt, but not as strongly as for water.

### NAc inactivation abolished sucrose and water intake, even during hunger and thirst

Since we observed that NAc inactivation almost fully abolished water, but not salt intake, we next examined the effects of NAc inactivation on food intake during hunger, and later also assessed the effects of NAc inactivation on water intake during thirst. We used the same experimental design as we had used to assess salt appetite, but instead monitored the intake of a 5% sucrose solution in the home cage after food restriction (*n* = 6). As such, animals had the choice between a bottle of sucrose (which was delivered to the animal right after the infusion) and a bottle of tap water (which was already present in the home cage of the animals). Animals in the control state, who were *ad libitum* fed, had access to regular chow before and during the experiment.

**Figure 4:**
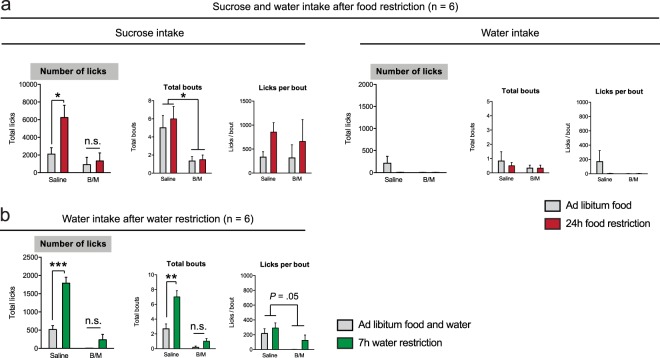
NAc inactivation reduced sucrose and water intake. (**a**) Sucrose (left) and water (right) intake was analyzed when animals were in a food restricted state (red) or in an *ad libitum* fed state (grey). A significant B/M × food restriction interaction effect on the number of licks for sucrose was found. Post-hoc tests Sidak’s test revealed a significant increase in the number of licks due to food restriction after saline infusion (t_5_ = 4.77, *P* = 0.010), but not after B/M infusion (t_5_ = 0.48, *P* = 0.877). A decrease in the number of licking bouts was found, which indicates that the interaction effect was mainly driven by a decrease in motivation for sucrose. Food restriction increased the number of licks for sucrose, driven by an increase in licks per bout. Water intake was extremely low and no significant effects could be detected on water licking behavior. (**b**) Water licking behavior was analyzed after water restriction. Water restriction increased the number of water licks, driven by an increase in number of licking bouts. NAc inactivation decreased overall water intake, as a significant main effect of B/M on the number of licks and the number of bouts were detected, as well as a trend towards a main effect of licks per bout. A significant B/M × water restriction interaction effect was observed of the number of licks for water which was driven by an increase in licking after saline infusion (t_5_ = 10.29, *P* = 0.0003) but not after B/M infusion (t_5_ = 1.87, *P* = 0.23) as revealed by post-hoc Sidak’s tests. There was also a significant B/M × water restriction interaction effect on the number of bouts, driven by an increase in bouts after saline (t_5_ = 7.24, *P* = 0.0016), but not B/M (t_5_ = 1.39, *P* = 0.40) infusion. A single asterisk annotation per graph indicates a main effect of B/M; a dual asterisk annotation in a graph denotes significance after post-hoc Sidak’s test (performed because a significant B/M × restriction interaction was found); see also the Supplementary statistics table. ***P < 0.001, **P < 0.01, *P < 0.05

We observed a significant B/M × food restriction interaction effect on the number of licks the animals made for sucrose (Fig. 4a, left panels). Post-hoc tests indicated that this was driven by a significant increase in the number of licks for sucrose upon food restriction after saline infusion, but not after B/M infusion. This effect seemed mainly driven by a decrease in the number of licking bouts, as we observed a main effect of B/M on this parameter, but not on the number of licks per bout. In contrast to baseline sucrose consumption, the total intake of water was extremely low (Fig. 4a, right panels), perhaps because the animals’ water homeostasis was relatively normal (compared to after sodium deprivation) and the animals had continuous access to water. Together, these data demonstrate that NAc inactivation reduced consumption of sucrose, and that this is independent of the energy balance of the animal.

Similar effects of NAc inactivation were observed on the intake of water during thirst (Fig. 4b). Water restriction increased the consumption of water (main effect of restriction on licks and the number of licking bouts), and B/M infusion into the NAc decreased overall water intake (significant main effect of B/M on number of licks and number of bouts; trend towards a main effect on the licks per bout). Furthermore, a significant B/M × water restriction interaction effect was observed on the number of licks for water and the number of licking bouts, which was driven by an increase in licking after saline infusion but not after B/M infusion. This indicates that pharmacological inactivation of the NAc abolished water intake, even when animals were thirsty.

### DA receptor antagonism in the nucleus accumbens does not alter salt appetite

**Figure 5:**
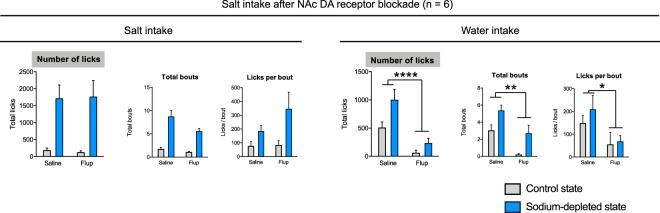
The effects of infusion of the DA receptor antagonist α-flupenthixol (Flup) on salt (left) and demineralized water (right) intake in rats in a sodium-depleted (blue) and control (grey) state. Infusion of α-flupenthixol did not affect total salt intake, nor the number of bouts or the number of licks per bout. Water intake was significantly decreased by infusion of the DA receptor antagonist, driven by decreases in both the number of bouts and licks per bout. A single asterisk annotation per graph indicates a main effect of B/M; see also the Supplementary statistics table. ****P < 0.0001, **P < 0.01, *P < 0.05

We next repeated the salt intake experiment in these animals (*n* = 6), but now infused the DA receptor antagonist α-flupenthixol into the NAc (25 µg/side), to study the importance of DAergic neurotransmission in the NAc for salt appetite. We observed that α-flupenthixol infusion did not affect salt intake, nor did it affect the number of licking bouts or licks per bout (Fig. 5, left panels). However, we did observe a significant effect of α-flupenthixol infusion on water intake (driven by both a decrease in the number of licking bouts and the size of these licking bouts; Fig. 5, right panels). These data suggest that the suppressing effect of pharmacological inactivation of the NAc on salt intake under sodium-depleted conditions is not driven by DAergic neurotransmission.

## Discussion

It is generally assumed that deprivation of a reward increases the motivation to obtain it, ranging from food^23^ to social behavior^24^ and drugs^25^. However, that a certain food can act either as punisher or as a reinforcer dependent on the homeostatic state of an organism is, to our knowledge, unique to salt. In our study, we demonstrated that VTA DA neurons in rats encode the appreciation of a salty solution, dependent on the animal’s internal sodium levels. As such, tasting salt under normal circumstances resulted in a suppression of licking and sub-baseline levels of DA neuron activity, indicating that this solution was considered aversive. Conversely, salt tasting after sodium depletion evoked vigorous licking for the solution, along with peaks in DA neuron activity that were even larger than the peaks previously observed during sucrose tasting. This finding is consistent with a recent study that demonstrated altered DA release in the nucleus accumbens shell in response to a NaCl solution after sodium deprivation^21^. Another study^22^ recently showed that sodium deprivation did not affect baseline activity of VTA DA neurons, *ex vivo* nor *in vivo*, in accordance with our finding that c-Fos expression was not altered in midbrain DA nuclei after furosemide treatment. A methodological consideration here is that we may have missed any acute effects of salt deprivation on c-Fos expression, since the peak in c-Fos expression lies between 60 and 120 minutes after a certain manipulation^26^ and our measurement was conducted after 48 hours of deprivation. However, a recent paper found that the same furosemide treatment as used in the current work resulted in strong c-Fos expression after 24 hours of salt deprivation^21^. Earlier work by Geerling *et al*. shows that even after 8 days of salt deprivation, responsive neurons still show increased c-Fos immunoreactivity^27^, perhaps signaling a need state for sodium. This indicates that the chronic effects can be detected if present, and that the lack of altered c-Fos expression upon salt deprivation is most probably due to unaffected baseline activity of midbrain DA neurons. Taken together, the findings from the photometry and c-Fos experiment suggest that sodium deprivation does not simply disinhibit the whole DA system, but that the changes in DA neuron activity are dependent on presentation of the salient salt solution. Thus, the DA system gets engaged upon presentation of the salient (salt) stimulus.

To subsequently investigate which output areas are involved in the regulation of salt appetite, we assessed the effects of inhibiting the two main output regions of the VTA, the NAc and the mPFC, on salt appetite. Several studies have suggested that during free-intake paradigms, the frequency of licking bouts (i.e. how often the animal initiates drinking) is a measure of incentive salience, or the motivation to obtain reward, whereas the bout size (i.e. the length of a drinking period) informs about the hedonic impact, or appreciation of reward^28–31^. We therefore performed a microstructure analysis of salt licking behavior after pharmacological inactivation of the NAc and demonstrated that the decrease in salt intake was driven by a reduction in the number of licking bouts, but not the size of these bouts, suggesting that the motivation for salt under a situation of salt deprivation was decreased upon NAc inactivation. To assess if this decrease in motivation for salt upon NAc inactivation was also true for other appetites, we investigated sucrose or water intake during food deprivation or water deprivation, respectively. The finding of decreased motivation was replicated in the sucrose intake experiment, where we observed that NAc inactivation reduced the number of sucrose licking bouts, but not the number of licks within these bouts. Together, this suggests that the motivational aspect of salt appetite is reduced by inactivation of the NAc, just as is the motivation for sugar when food restricted. In contrast to these findings, NAc inactivation did have a significant effect on the size of the licking bouts for water, a liquid that has a neutral taste, suggesting that the absence of an effect of NAc inactivation on the sucrose and salt licking bout size is related to its taste, and that taste acts as the conditioned stimulus for the homeostatic need. In fact, NAc inactivation almost fully suppressed water intake in all of the experiments, even when the animals were thirsty. The observation that this was not the case for salt and sucrose consumption suggests that the attenuated water intake was not the result of a general behavioural impairment, for example because of motor deficits. Interestingly, it has been shown previously that pharmacological inactivation of the medial shell subregion of the NAc leads to an increase in palatable food intake^32^. These findings are difficult to reconcile with the findings from the current study, but may be related to the larger infusion volume that we have used (1 µl). This was used in order to inactivate the entire NAc, rather than just the medial shell^33^. Indeed, it is well known that the core and shell subregions of the NAc play distinct, complementary roles in the pursuit, consumption and appreciation of palatable food, which likely explains the different effects of inactivating the medial shell versus entire NAc^32,34,35^. In contrast to inactivation of the NAc, we found no effects of inactivation of the mPFC on salt appetite.

Since we found that VTA DA neurons encode the appreciation of salt dependent on homeostatic state of the animal and that inactivation of the NAc decreased the motivational aspects of salt appetite, we finally inhibited DA receptors locally within the NAc to study the role of NAc DAergic neurotransmission in salt appetite. After pharmacological blockade of NAc DA receptors using α-flupenthixol, we observed no effects on salt appetite. Interestingly, we did again observe an effect of the DA receptor antagonist on water intake during this experiment, just as after inactivation of the NAc with B/M. This suggests that the motivation for salt does not require NAc DA, but that this is not necessarily the case for other types of motivation.

The lack of effect of NAc DA receptor blockade on salt appetite is somewhat surprising, given that mesoaccumbens DA is considered a driving force behind motivation for rewards^36,37^. Furthermore, previous studies have reported alterations in the mesolimbic dopamine system and its inputs after sodium deprivation, both morphologically^20^ and functionally^21^. Our findings are not necessarily conflicting with these data, as we also showed that VTA DA neuron dynamics during salt and sucrose tasting are dependent on the sodium balance of the animal. This suggests altered reward processing after sodium deprivation, which may logically also affect downstream DA release and hence morphological and structural changes to downstream areas. However, we do show that mesolimbic DA neurotransmission is not *necessary* for the behavioral expression of salt appetite.

That said, the finding that a switch in salt appreciation upon a change in body sodium levels is encoded by VTA DA neurons, but that blockade of DA receptors in one of its most important downstream regions does not hamper salt appetite may seem counterintuitive. A possible explanation is that behavioral adaptation to a shortage in sodium is so crucial, since it can be a prerequisite for survival, that it is redundantly coded in the brain, and thus relies on a variety of brain regions. For example, the VTA also projects to the subthalamic nucleus, which projects to the substantia nigra and the ventral pallidum, which again sends efferents to the substantia nigra, lateral hypothalamus, lateral pre-optic area, pedunculopontine nucleus, and brainstem^38^. All these regions form a complex network of the ventral basal ganglia, which could function as backup for dysfunction of the NAc. Furthermore, the VTA is known to directly project to the lateral hypothalamus, also a key region of the reward system, which forms a neural circuit with the parabrachial nucleus and the nucleus of the solitary tract, which has been shown to be involved in the sensory and motor aspects of feeding^39,40^.

Whether the apparent independence of sodium appetite of NAc DA extends towards other types of motivated behaviors, such as food intake and social behaviors remains an important question. Acute pharmacological NAc DA receptor blockade has been shown to decrease social play behavior in rats, but only when animals are highly motivated to do so (i.e., after social isolation)^24^. Likewise, it reduces sucrose self-administration when the response requirement increases within a session (i.e., a progressive ratio schedule of reinforcement), but not free chow intake^41^. It is therefore thought that NAc DA is important for certain types of motivated behaviors, most prominently those involving explicit effort or reward-based learning^35,37,42^. Importantly, in our task, the salt solution can be consumed freely and does not involve any learning processes, since the animals had already been exposed to the salty solution before. It is therefore important to note that our conclusions only apply to the general increase in effort-free salt intake that is observed under sodium-depleted conditions.

In sum, we have used a multidisciplinary approach, including c-Fos immunohistochemistry, fiber photometry and behavioral pharmacology, to assess the role of the mesocorticolimbic DA system in salt appetite. We have substantiated findings from earlier studies regarding the role of VTA neurons in salt appetite, and provide novel insights into the role of its target regions in this behavior. We show that the NAc, but not mPFC, is essential for the behavioral expression of salt appetite by mediating its motivational, but not hedonic, component. This role of the NAc in salt appetite is independent of DA, although we show that DA neurons themselves do encode the appreciation of salt.

## Methods

### Animals

All experiments were approved by the Animal Ethics Committee of the Utrecht University, and were conducted in agreement with Dutch (Wet op de Dierproeven, revised 2014) and European regulations (Guideline 86/609/EEC; Directive 2010/63/EU).

A total of 46 male rats were used in the experiments. Male Long-Evans rats (Rj:Orl; Janvier Labs, France) were used for the micro-infusion experiments (*n* = 14), male Wistar rats (Crl:WU; Charles River, Germany) were used for c-Fos analysis (*n* = 22), and TH::Cre transgenic rats (bred in-house by crossing heterozygous TH::Cre+/− male rats with wild type Rj:Orl mates) were used for fiber photometry (*n* = 6 TH::Cre+ injected with DIO-GCaMP6s and, *n* = 4 TH::Cre− injected with eYFP). All rats weighted ~250 g at the start of the experiments, were individually housed under controlled temperature (20 °C) conditions, with a 12 h light/dark cycle (lights off at 7:00 a.m.), and received a wood block as cage enrichment. When not being tested, animals had *ad libitum* access to demineralized water and a 0.45 M sodium chloride solution (or a 5% sucrose solution, prior to the sucrose intake experiment) and standard chow (Special Diet Service, United Kingdom) in the home cage. Preceding sodium intake test days, animals were salt deprived, during which they only had access to demineralized water and a sodium-deficient chow (Teklad Custom Diet, Envigo, United States). Preceding the sucrose intake test days, animals were food restricted, during which they had no access to chow or the sucrose solution for 24 h.

### Surgeries

Anesthesia was induced using a mixture of 0.315 mg/kg fentanyl and 10 mg/kg fluanisone (Hypnorm, Janssen Pharmaceutica, Belgium) that was injected intramuscularly. Animals were placed in a stereotaxic apparatus (David Kopf Instruments, USA) and an incision was made along the skull midline.

For fiber photometry experiments, the same surgical procedure was applied as described previously^15^. In brief, TH::Cre rats were injected with 1 μl of AAV5-FLEX-hSyn-GCaMP6s (University of Pennsylvania Vector Core) at a titer of 1 × 10^12^ particles/ml unilaterally into the right VTA (−5.40 mm AP, ± 2.20 mm ML from Bregma, at an angle of 10°, and −8.90 mm DV from the skull). A 400 µm implantable fiber was lowered to 0.1 mm above the injection site and attached with dental cement.

For micro-infusion experiments, 26-gauge stainless steel guide cannulas (Plastics One, USA) were implanted above the NAc (two single cannulas; +1.20 mm anteroposterior (AP), ±2.80 mm mediolateral (ML) from Bregma, at an angle of 10°, and the guide was lowered to −6.80 mm dorsoventral (DV) from the skull) or the mPFC (one double cannula with a width of 1.2 mm; +3.20 mm AP, ± 0.60 mm ML from Bregma, and the guide was lowered to −2.60 mm DV from the skull). Cannulas were secured to the skull with screws and dental cement, and dummy injectors were placed inside the cannulas to prevent blockage. Single injectors for the NAc protruded 0.5 mm beyond the guides (targeting −7.30 mm DV from the skull) and double injectors for the mPFC protruded 1 mm beyond the guides (targeting −3.50 mm DV from the skull).

To prevent dehydration of the rats, they were given 10 mL of saline subcutaneous (s.c.) after surgery. Starting on the day of surgery, rats were given carprofen as analgesia (s.c. injection of 5 mg/kg per day for 3 days). All rats were allowed to recover from surgery for at least 7 days before behavioral testing began.

### Sodium deprivation

The sodium deprivation protocol has been adapted from ref.^21^. Before the sodium deprivation procedure, all cages were cleaned to prevent the rats from repleting their sodium levels by eating their own feces. Sodium depletion was induced by an s.c. injection of the diuretic drug furosemide (20 mg/kg dissolved in sterile H_2_O, given in 2 injections of 10 mg/kg 1 hour apart). Control animals received s.c. saline injections. In the 24 h that followed, sodium-depleted animals received sodium-free chow (Teklad Custom Diet, Envigo, United States), and control animals received regular chow (Special Diet Service, United Kingdom). In the first three hours after the first furosemide injection, animals had no access to water, to confirm success of the procedure by observing a body weight loss. After these 3 hours, all animals received demineralized water, which was especially heavily consumed by the animals that were previously injected with furosemide. 24 hours after the first furosemide injection, animals were given a bottle containing a 0.45 M NaCl solution, and intake of this solution (as well as intake of the demineralized water, which was already present in the cage) was monitored for 1 h using mechanical lickometers that were present in the home cage. Animals were always tested in a counterbalanced fashion, so that half of the animals were first tested in a control state, i.e., 24 h after s.c. saline injection, and the other half in a sodium-depleted state, i.e., 24 h after s.c. furosemide injection. Drinking behavior was assessed as cumulative intake (number of licks), number of licking bouts, and licks per licking bout for both the intake of demineralized water and the 0.45 M NaCl solution. A minimum of 5 licks was considered a bout, which ended when the animals did not lick for at least 1 min. Bout analysis was performed in Matlab R2014a (MathWorks Inc., United States).

### *In vivo* fiber photometry

Technical details about our fiber photometry setup have been published elsewhere^15^. In brief, animals were injected with a Cre-dependent GCaMP6s in the right VTA, and a 400 µm fiber was secured 0.1 mm dorsal of the injection site. Animals were connected to a 400 µm core fiber optic patch cable through which lock-in amplified blue LED light was delivered. Emission light was captured with a photoreceiver, digitized, and dF/F_0_ values were computed with F_0_ being defined as the mean of the middle 50% of values in the 30 seconds before each time point F.

Each rat was tested on the behavioral task twice, once in a salt-depleted state and once in a control state, and the task was conducted in operant conditioning chambers (Med Associates, USA). The chambers were equipped with one optical lickometer (delivering both solutions through the same spout), and on the other side of the chamber a house light and auditory tone generator. All animals were food restricted for 24 h before the measurement, to increase the motivation for sucrose (making sure the animals lick during every trial).

In the task, a 5-second tone initiated the trial, and the first lick after tone offset triggered the fluid pump, which delivered a droplet of the solution over a period of 5 s. If the animal did not make a lick within 5 s after tone offset, no reward was obtained and the inter-trial interval of 30 s commenced. If the animal did make a lick within 5 s after tone offset, the pump delivered a 0.88 M sucrose solution in 75% of the trials and a 0.30 M NaCl solution in 25% of the trials (in random order). After the 5-second liquid delivery, a 30-second inter-trial interval separated the current trial from the onset of the next trial. No cue lights were used and the house light was turned on continuously to prevent the signal to be contaminated by lights from the environment. Individual trial responses were time-locked to the 5-second tone that started the trial and mean dF/F of trial responses to sucrose and of trial responses to sodium was calculated. The number of licks during the trials was assessed using the lickometers that were monitored by MedPC software. The task continued until the animal had made at least 80 trials.

### Microinfusions

For the infusion experiments, n = 7 (NAc; all experiments performed in the same animals. see Fig. S3c) and n = 8 (mPFC) rats were used. Animals were habituated to the infusion procedure by infusing saline (0.5 μl/side) the day before the first experiment. Rats were brought into a salt depleted state or in a control state, as described in the paragraph above, and 24 h later they received infusions with saline (1 μl/side for the NAc, 0.5 μl/side for the mPFC) or a mixture of baclofen (1nmol; Sigma-Aldrich, The Netherlands) and muscimol (0.1 nmol; Sigma-Aldrich, The Netherlands) dissolved in saline (1 μl/side for the NAc, 0.5 μl/side for the mPFC; based on the spread observed in ref.^33^). Furosemide vs saline injections and baclofen-muscimol vs saline infusions were performed in a Latin Square repeated measures design (each animal was tested 4 times). Testing of a single experiment was performed across ~2 weeks, as we allowed the animals to recover after every of the four measurements for at least 48 hours.

Drugs were infused at a rate of 0.5 μl/min, and the injectors were left in place for an additional 30 s after the infusion was complete to allow for diffusion of saline/baclofen-muscimol into the brain. After the infusion procedure, animals were placed back into their home cage, and a bottle with a 0.45 NaCl solution was given 5 minutes later. In the dopamine receptor antagonist infusion experiment, we used the same experimental procedure as in the pharmacological inactivation experiments, except that 25 µg of cis-(Z)-α-flupenthixol dihydrochloride (Sigma-Aldrich, The Netherlands) was infused, dissolved in 1 µl saline.

### c-Fos analysis

For c-Fos analysis, 11 animals were brought into a salt-deprived state as described in the procedure above, and 11 animals were used as control animals. 48 hours after the first furosemide injection, all 22 rats received an i.p. injection of sodium pentobarbital and perfused with phosphate-buffered saline (PBS) followed by 4% paraformaldehyde (PFA) in PBS. After extraction, brains were post-fixated in 4% PFA in PBS at 4 °C for 24 h, and stored in a 30% sucrose in PBS solution at 4 °C. For immunohistochemical quantification of the number of activated neurons, brains were stained for the immediate early gene c-Fos. Brain slices (50 μm) were blocked in 3% normal goat serum (NGS) and 0.5% Triton-X-100 in PBS. Slices were incubated overnight in primary antibody rabbit anti-c-Fos (1:1000, Cell Signaling) in 3% NGS in PBS at room temperature. Subsequently, slices were incubated for 2 h in biotinilated antibody goat anti-rabbit (1:200, Vector labs) in 3% NGS in PBS, and afterwards in Biotin/Avidin (1:1000, Vectastain) in PBS for 1 h. This complex was visualized by exposing the slices for 5 min to a solution of liquid DAB (3,3’-Diaminobenzidine, Dako) and 10% nickel ammonium sulphate. All sections were dehydrated using increasing series of ethanol, cleared in xylene and coverslipped with Entallan (Merck Millipore). Sections were photographed by a brightfield microscope with a 10X lense (AxioImager M2). Slices comprising the VTA were manually aligned in Adobe Illustrator, and ImageJ (Version 1.48 v) was used to extract the coordinates of c-Fos positive neurons by applying a bandpass filter over the Fourier-transformed image, followed by a search for maximum intensity points. Heatmaps of c-Fos expression were generated based on the coordinates of the c-Fos positive cells using MATLAB (The MathWorks Inc., version R2014a).

### Exclusion criteria

One animal was excluded from c-Fos analysis because brain slices were not of sufficient quality. One additional animal was excluded from c-Fos analysis because the brain slices did not show any expression. One animal was excluded from the sucrose intake (after food restriction) experiment, water intake (after water deprivation) experiment and dopamine receptor antagonist infusion experiment, because it was suspected to develop diabetes (it drank excessive amounts of water and sucrose water, and the bedding was continuously wet).

### Data analysis and statistics

Data analysis was performed with MATLAB, statistical analysis using GraphPad Prism (GraphPad Software Inc., version 6.0). Statistical comparisons were made using a two-tailed t-test for a single comparison (c-Fos experiment) and a (repeated measures) ANOVA was used for multiple comparisons (all other experiments), followed by a t-test with Šidák’s multiple comparisons correction when a significant interaction effect (*P* < 0.05) was found between the two factors of the ANOVA. Bar graphs represent the mean ± standard error of the mean. In all figures: ns not significant, *P < 0.05, **P < 0.01, ***P < 0.001, ****P < 0.0001.

## Supplementary information

Supplementary Dataset 1

## Data Availability

The datasets generated during the current study are available from the corresponding author on reasonable request.
