# Supplementary material for: Limbic control over the homeostatic need for sodium
Source: Sci Rep. 2019 Jan 31;9:1050. doi: 10.1038/s41598-018-37405-w (PMC6355778; doi:10.1038/s41598-018-37405-w)
Supplement: Supplementary file 1 — Supplementary Dataset 1 [file 41598_2018_37405_MOESM1_ESM.pdf]

Supplement

Limbic control over the homeostatic need for sodium

Jeroen P.H. Verharen[1,2,4]
Theresia J.M. Roelofs[1,4]
Shanice Menting-Henry[1]
Mieneke C.M. Luijendijk[1]
Louk J.M.J. Vanderschuren[2]
Roger A.H. Adan[1,3,*]

[1] Brain Center Rudolf Magnus, Department of Translational Neuroscience, University Medical Center Utrecht, Utrecht, The Netherlands.

[2] Department of Animals in Science and Society, Division of Behavioural Neuroscience, Faculty of Veterinary Medicine, Utrecht University, Utrecht, The Netherlands.

[3] Institute of Physiology and Neuroscience, Sahlgrenska Academy at the University of Gothenburg, Gothenburg, Sweden.

[4] These authors contributed equally.

* Corresponding author: r.a.h.adan@umcutrecht.nl.

Supplementary Figure 1

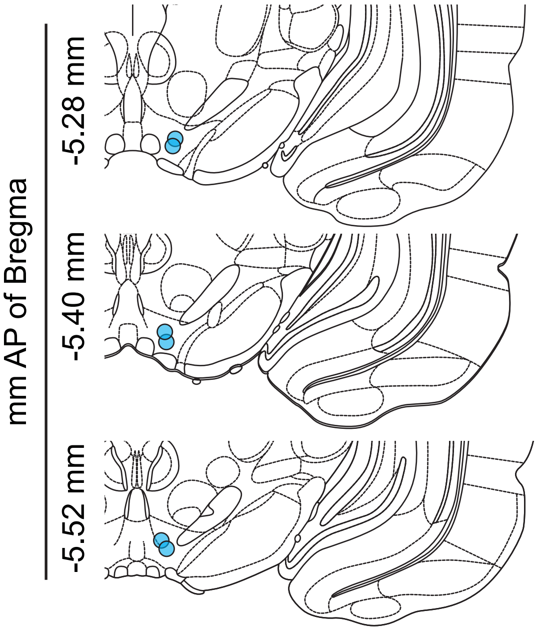

Supplementary Figure 1
Fiber placements of photometry experiments

Supplementary Figure 2

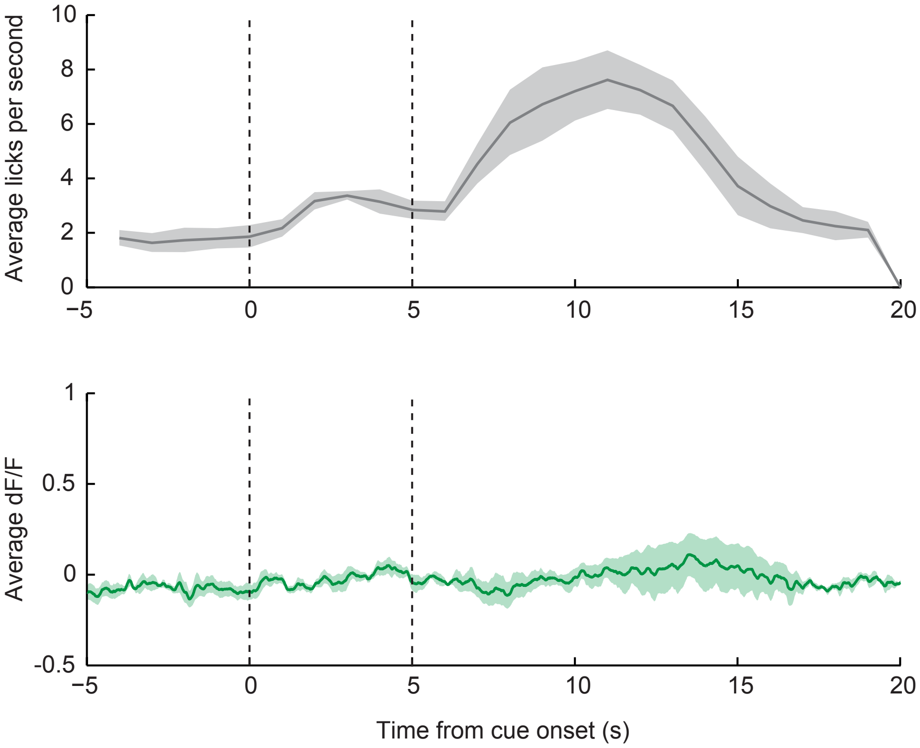

Supplementary Figure 2 In vivo fiber photometry of VTA neurons from animals injected with a YFP control fluorophore. An activity-independent control fluorophore was injected into the VTA of control animals (n=4) and in vivo fiber photometry indicated no changes in fluorescent activity in these controls (lower panel). Upper panel shows the average licking rate of the animals for sucrose reward. Line and shading represent mean and standard error of the mean, respectively.

Supplementary Figure 3

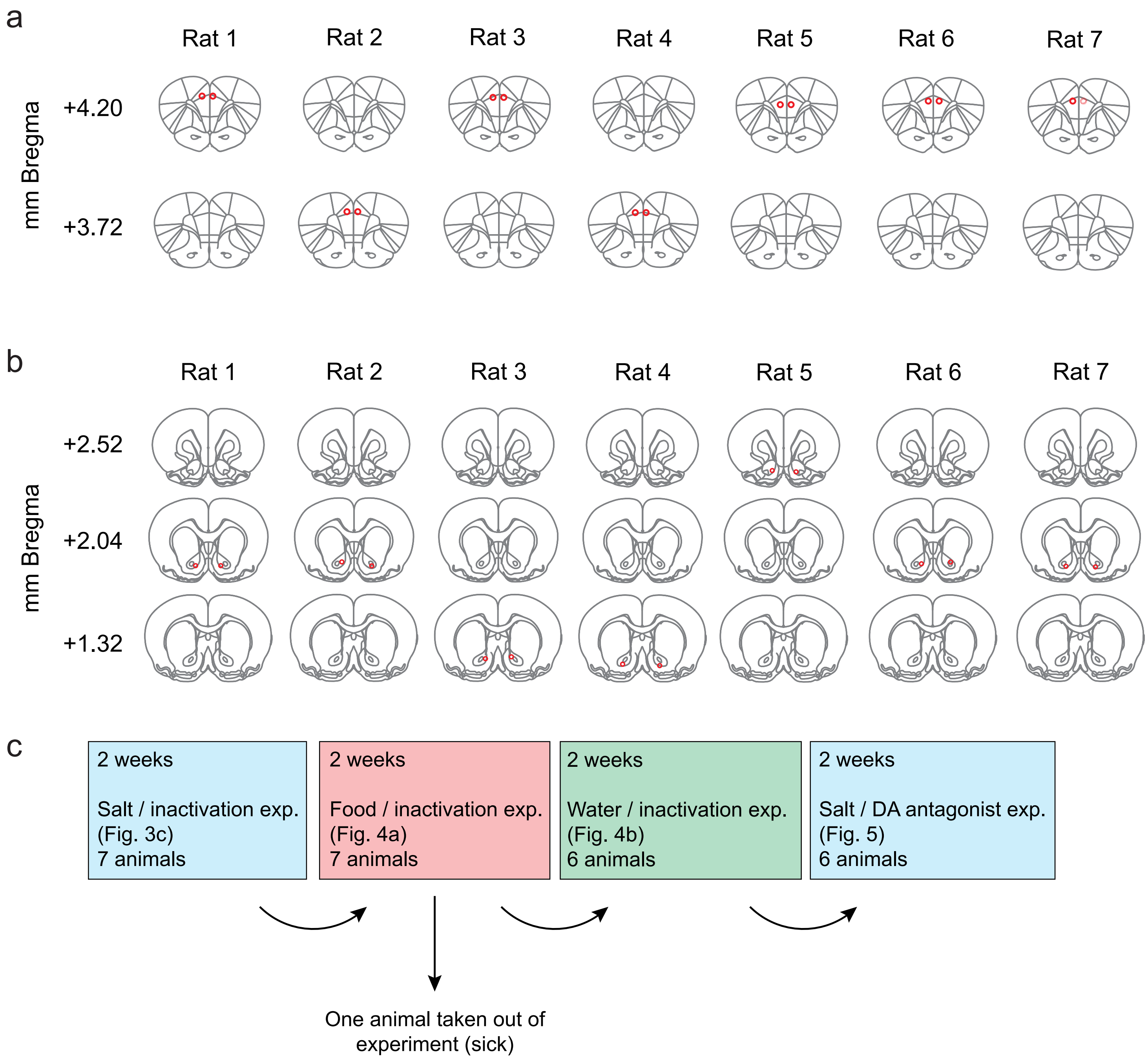

Supplementary Figure 3 Histological verification of guide cannula placement. Correct placement of the guide cannulas used for local infusions was verified for all animals in which the mPFC (a) or the NAc (b) was targeted. Rat 1 from (b) was excluded from figures 4 and 5 because it developed diabetes. (c) Timeline of experiments for animals with cannulas in the NAc. In each block of 2 weeks, animals were tested four times in the free-intake paradigm (in a counterbalanced, semi-random design).

Supplementary statistics table

P values of 2-way repeated measures ANOVA on the outcome parameters of the free-intake assay

	ANOVA factor	Salt intake			Water intake			Sucrose intake		
		# of licks	# of bouts	Licks per bout	# of licks	# of bouts	Licks per bout	# of licks	# of bouts	Licks per bout
Fig. 3b	B/M	*P* = .30	*P* = .42	*P* = .38	*P* = .58	*P* = .91	*P* = .12			
	State	**_P_ < .001**	**_P_ < .001**	**_P_ < .01**	*P* = .06	**_P_ = .04**	*P* = .90			
	B/M × State	*P* = .80	*P* = .10	*P* = .31	*P* = .89	*P* = .51	*P* = .69			
		# of licks	# of bouts	Licks per bout	# of licks	# of bouts	Licks per bout	# of licks	# of bouts	Licks per bout
Fig. 3c	B/M	**_P_ < .01**	**_P_ = .04**	*P* = .59	**_P_ = .04**	**_P_ = .01**	**_P_ < .01**			
	State	*P* = .06	**_P_ = .02**	*P* = .08	*P* = .14	*P* = .17	*P* = .24			
	B/M × State	*P* = .15	*P* = .20	*P* = .74	*P* = .13	*P* = .16	*P* = .23			
		# of licks	# of bouts	Licks per bout	# of licks	# of bouts	Licks per bout	# of licks	# of bouts	Licks per bout
Fig. 4a	B/M				*P* = .22	*P* = .24	*P* = .32	*P* = .05	**_P_ = .018**	*P* = .80
	Restriction				*P* = .24	*P* = .75	*P* = .33	**_P_ = .013**	*P* = .48	**_P_ = .031**
	B/M × Restriction				*P* = .24	*P* = .68	*P* = .34	**_P_ = .029**	*P* = .71	*P* = .48
								*post-hoc tests performed **		
		# of licks	# of bouts	Licks per bout	# of licks	# of bouts	Licks per bout	# of licks	# of bouts	Licks per bout
Fig. 4b	B/M				**_P_ = .0016**	**_P_ = .0025**	**_P_ = .05**			
	Restriction				**_P_ = .0015**	**_P_ = .0038**	*P* = .18			
	B/M × Restriction				**_P_ = .0019**	**_P_ = .0090**	*P* = .66			
					*post-hoc tests performed **	*post-hoc tests performed **				
		# of licks	# of bouts	Licks per bout	# of licks	# of bouts	Licks per bout	# of licks	# of bouts	Licks per bout
Fig. 5	Flup	*P* = .97	*P* = .09	*P* = .16	**_P_ < 0.0001**	**_P_ = .0026**	**_P_ = .03**			
	State	**_P_ = .011**	**_P_ = .0002**	*P* = .07	*P* = .09	**_P_ = .0081**	*P* = .51			
	Flup × State	*P* = .76	*P* = .15	*P* = .25	*P* = .31	*P* = .90	*P* = .69			

** a post-hoc Sidak's test was performed when the ANOVA yielded a significant (P < 0.05) interaction effect*
